# Menthyl 2-oxo-2*H*-chromene-3-carboxyl­ate

**DOI:** 10.1107/S1600536809035995

**Published:** 2009-09-12

**Authors:** Cui-Lian Xu, Shan-Yu Liu, Gang Chen, Guo-Yu Yang, Ming-Qin Zhao

**Affiliations:** a College of Sciences, Henan Agricultural University, Zhengzhou 450002, People’s Republic of China; b College of Tobacco Science, Henan Agricultural University, Zhengzhou 450002, People’s Republic of China

## Abstract

The title compound, C_20_H_24_O_4_, was synthesized from the reaction of 2-oxo-2*H*-chromene-3-acyl chloride and menthol. The mean plane of the ester group and that of the four essentially planar (maximum deviation 0.0112 Å) C atoms of the chair-form cyclo­hexyl ring form dihedral angles of 43.8 (3) ° and 81.8 (1)°, respectively, with the mean plane of the coumarin ring system. In the crystal structure, weak inter­molecular C—H⋯O hydrogen bonds connect the mol­ecules into a two-dimensional network.

## Related literature

For the applications of coumarin compounds, see: Yu *et al.* (2003 ▶, 2007 ▶).
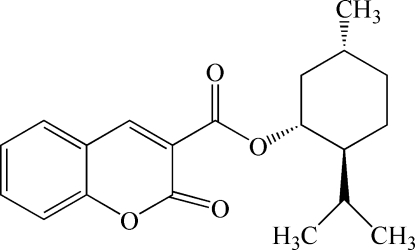


## Experimental

### 

#### Crystal data

C_20_H_24_O_4_
*M* *_r_* = 328.39
Orthorhombic, 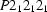

*a* = 11.080 (2) Å
*b* = 12.408 (3) Å
*c* = 13.532 (3) Å
*V* = 1860.3 (6) Å^3^
*Z* = 4
Mo *K*α radiation
μ = 0.08 mm^−1^
*T* = 291 K
0.18 × 0.18 × 0.17 mm

#### Data collection

Rigaku R-AXIS-IV diffractometer
Absorption correction: multi-scan (*ABSCOR*; Higashi,1995 ▶) *T*_min_ = 0.986, *T*_max_ = 0.986
5812 measured reflections
1943 independent reflections
1654 reflections with *I* > 2σ(*I*)
*R*_int_ = 0.063

#### Refinement

*R*[*F* ^2^ > 2σ(*F* ^2^)] = 0.057
*wR*(*F* ^2^) = 0.138
*S* = 1.09
1943 reflections
218 parameters
H-atom parameters constrained
Δρ_max_ = 0.15 e Å^−3^
Δρ_min_ = −0.17 e Å^−3^

### 

Data collection: *R-AXIS* (Rigaku, 1997 ▶); cell refinement: *R-AXIS* data reduction: *R-AXIS*; program(s) used to solve structure: *SHELXS97* (Sheldrick, 2008 ▶); program(s) used to refine structure: *SHELXL97* (Sheldrick, 2008 ▶); molecular graphics: *TEXSAN* (Molecular Structure Corporation & Rigaku (2000 ▶) and *PLATON* (Spek, 2009 ▶); software used to prepare material for publication: *TEXSAN*.

## Supplementary Material

Crystal structure: contains datablocks I, global. DOI: 10.1107/S1600536809035995/lh2883sup1.cif

Structure factors: contains datablocks I. DOI: 10.1107/S1600536809035995/lh2883Isup2.hkl

Additional supplementary materials:  crystallographic information; 3D view; checkCIF report

## Figures and Tables

**Table 1:** Hydrogen-bond geometry (Å, °)

*D*—H⋯*A*	*D*—H	H⋯*A*	*D*⋯*A*	*D*—H⋯*A*
C3—H3*A*⋯O2^i^	0.93	2.43	3.288 (5)	154
C5—H5*A*⋯O3^ii^	0.93	2.42	3.276 (5)	152

Symmetry codes: (i) 
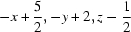
; (ii) 
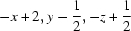
.

**Fig. 1.**
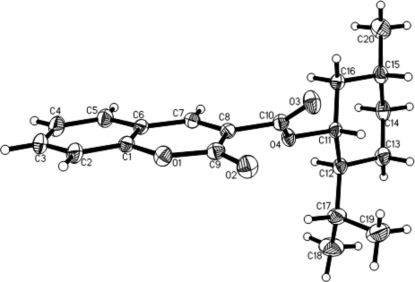
The molecular structure of the title compound showing the atom numbering scheme. Displacement ellipsoids are drawn at the 30% probability level.

**Fig. 2.**
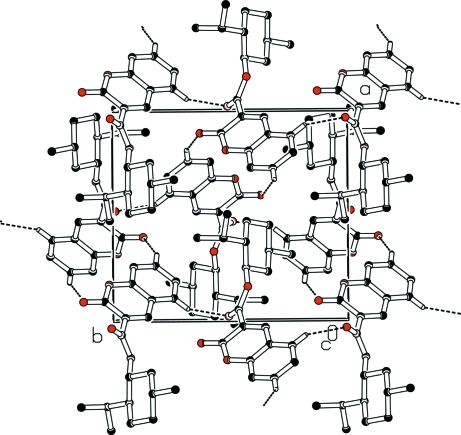
Part of the crystal structure of the title compound showing weak C– H···O hydrogen bonds as dashed lines. Only H atoms involved in H– bonding have been shown.

## supplementary crystallographic information

### Comment

Coumarins are a type of plant-derived compounds, which are of interest mainly because of their excellent bioactivities in many areas (Yu *et al.*, 2003; Yu *et al.*, 2007). Some coumarin derivatives have shown to be potential anti-HIV agents, antibiotics, and antioxidants. We have synthesized the title compound (I) and its crystal structure is reported herein.

The molecular structure of (I) is shown in Fig. 1. The compound is composed of a coumarin core with a menthyloxycarbonyl in 3-position. The dihedral angle between the plane of ester group and the plane of coumarin ring system is 43.8 (3)°. The dihedral angle between the coumarin ring system and the plane defined by four essentially planar carbon atoms (C11/C13/C14/C16) of the chair form cyclohexyl ring is 81.8 (1)°. In the crystal structure, weak intermolecular C—H···O hydrogen bonds connect molecules into a two-dimensional network (Fig. 2).

### Experimental

A solution of menthol (0.0072 mol) dissolved in dried methyl dichloride (DCM) (25ml) was added dropwise to a solution of 2-oxo-2*H*-chromene -3-acyl chloride (0.0072 mol) dissolved in DCM (25 ml) and triethylamine (1 ml) at room temperature. The reaction mixture was stirred for 24 h (mornitored by TLC). The mixture was then neutralized with 5% HCl and washed with saturated NaHCO_3_ and brine respectively. The organic phase was dried over Na_2_SO_4_ and evaporated under the reduced pressure. The resulting residue was purified by column chromatography (EtOAc: petroleum ether) to give the pure compound. Single crystals of the title compound suitable for X-ray diffractions were obtained by slow evaporation of a mixed solvent (ethyl acetate: petroleum ether = 1:1, 10 ml) solution of the title compound (0.035 g).

### Refinement

In the absence of significant anomalous dispersion effects Friedel pairs were merged before refinement. The absolute configuration is based on that of the starting material. All H atoms were placed in caculated positions, with C—H = 0.93 Å, and *U*_iso_(H)=1.2U_eq_(C) for aromatic H atoms; C—H = 0.96 Å, and *U*_iso_(H)=1.5 *U*_eq_(C) for methy H atoms. The final difference map had a highest peak at 0.64 Å from atom O2 and a deepest hole at 1.60 Å from atom C3.

### Crystal data

**Table:**

C_20_H_24_O_4_	*F*(000) = 704
*M**_r_* = 328.39	*D*_x_ = 1.173 Mg m^−^^3^
Orthorhombic, *P*2_1_2_1_2_1_	Mo *K*α radiation, λ = 0.71073 Å
Hall symbol: P 2ac 2ab	Cell parameters from 489 reflections
*a* = 11.080 (2) Å	θ = 2.2–25.5°
*b* = 12.408 (3) Å	µ = 0.08 mm^−^^1^
*c* = 13.532 (3) Å	*T* = 291 K
*V* = 1860.3 (6) Å^3^	Prism, colorless
*Z* = 4	0.18 × 0.18 × 0.17 mm

### Data collection

**Table:**

Rigaku R-AXIS-IV diffractometer	1943 independent reflections
Radiation source: fine-focus sealed tube	1654 reflections with *I* > 2σ(*I*)
graphite	*R*_int_ = 0.063
Detector resolution: 0 pixels mm^-1^	θ_max_ = 25.5°, θ_min_ = 2.2°
Oscillation frames scans	*h* = −13→13
Absorption correction: multi-scan (*ABSCOR*; Higashi,1995)	*k* = 0→15
*T*_min_ = 0.986, *T*_max_ = 0.986	*l* = −16→16
5812 measured reflections	

### Refinement

**Table:**

Refinement on *F*^2^	Secondary atom site location: difference Fourier map
Least-squares matrix: full	Hydrogen site location: inferred from neighbouring sites
*R*[*F*^2^ > 2σ(*F*^2^)] = 0.057	H-atom parameters constrained
*wR*(*F*^2^) = 0.138	*w* = 1/[σ^2^(*F*_o_^2^) + (0.0742*P*)^2^ + 0.172*P*] where *P* = (*F*_o_^2^ + 2*F*_c_^2^)/3
*S* = 1.09	(Δ/σ)_max_ < 0.001
1943 reflections	Δρ_max_ = 0.15 e Å^−^^3^
218 parameters	Δρ_min_ = −0.17 e Å^−^^3^
0 restraints	Extinction correction: *SHELXL*, Fc^*^=kFc[1+0.001xFc^2^λ^3^/sin(2θ)]^-1/4^
Primary atom site location: structure-invariant direct methods	Extinction coefficient: 0.025 (5)

### Special details

**Table:**

Geometry. All e.s.d.'s (except the e.s.d. in the dihedral angle between two l.s. planes) are estimated using the full covariance matrix. The cell e.s.d.'s are taken into account individually in the estimation of e.s.d.'s in distances, angles and torsion angles; correlations between e.s.d.'s in cell parameters are only used when they are defined by crystal symmetry. An approximate (isotropic) treatment of cell e.s.d.'s is used for estimating e.s.d.'s involving l.s. planes.
Refinement. Refinement of *F*^2^ against ALL reflections. The weighted *R*-factor *wR* and goodness of fit *S* are based on *F*^2^, conventional *R*-factors *R* are based on *F*, with *F* set to zero for negative *F*^2^. The threshold expression of *F*^2^ > σ(*F*^2^) is used only for calculating *R*-factors(gt) *etc*. and is not relevant to the choice of reflections for refinement. *R*-factors based on *F*^2^ are statistically about twice as large as those based on *F*, and *R*- factors based on ALL data will be even larger.

### Fractional atomic coordinates and isotropic or equivalent isotropic displacement parameters (Å^2^)

**Table:**

	*x*	*y*	*z*	*U*_iso_*/*U*_eq_	
O1	1.1789 (2)	1.01848 (18)	0.10921 (18)	0.0682 (7)	
O2	1.0992 (3)	1.1284 (2)	0.2180 (2)	0.0863 (8)	
O3	0.9734 (2)	1.0101 (2)	0.37733 (18)	0.0852 (8)	
O4	0.83141 (18)	0.92908 (18)	0.28562 (15)	0.0591 (6)	
C1	1.1825 (3)	0.9219 (3)	0.0601 (2)	0.0593 (8)	
C2	1.2617 (3)	0.9135 (3)	−0.0176 (3)	0.0814 (11)	
H2A	1.3124	0.9706	−0.0337	0.098*	
C3	1.2651 (4)	0.8204 (4)	−0.0710 (3)	0.0929 (13)	
H3A	1.3179	0.8145	−0.1242	0.111*	
C4	1.1909 (5)	0.7341 (4)	−0.0468 (3)	0.0931 (14)	
H4A	1.1947	0.6707	−0.0833	0.112*	
C5	1.1114 (4)	0.7426 (3)	0.0315 (3)	0.0760 (10)	
H5A	1.0618	0.6849	0.0478	0.091*	
C6	1.1055 (3)	0.8379 (2)	0.0861 (2)	0.0545 (7)	
C7	1.0247 (3)	0.8555 (2)	0.1674 (2)	0.0547 (7)	
H7A	0.9738	0.8000	0.1873	0.066*	
C8	1.0208 (3)	0.9499 (2)	0.2151 (2)	0.0508 (7)	
C9	1.0994 (3)	1.0387 (3)	0.1849 (2)	0.0611 (8)	
C10	0.9412 (3)	0.9684 (3)	0.3017 (2)	0.0571 (8)	
C11	0.7426 (3)	0.9362 (2)	0.3663 (2)	0.0565 (8)	
H11A	0.7545	1.0039	0.4022	0.068*	
C12	0.6189 (3)	0.9375 (3)	0.3173 (2)	0.0656 (9)	
H12A	0.6123	0.8708	0.2789	0.079*	
C13	0.5222 (3)	0.9316 (3)	0.3989 (3)	0.0834 (11)	
H13A	0.5242	0.9976	0.4373	0.100*	
H13B	0.4431	0.9261	0.3685	0.100*	
C14	0.5413 (4)	0.8365 (3)	0.4669 (3)	0.0853 (12)	
H14A	0.4794	0.8369	0.5175	0.102*	
H14B	0.5327	0.7704	0.4293	0.102*	
C15	0.6634 (4)	0.8376 (3)	0.5156 (3)	0.0755 (10)	
H15A	0.6690	0.9033	0.5555	0.091*	
C16	0.7619 (3)	0.8429 (3)	0.4359 (3)	0.0668 (9)	
H16A	0.8401	0.8502	0.4675	0.080*	
H16B	0.7620	0.7761	0.3986	0.080*	
C17	0.5998 (4)	1.0313 (4)	0.2444 (3)	0.0888 (12)	
H17A	0.6684	1.0305	0.1987	0.107*	
C18	0.4854 (5)	1.0146 (6)	0.1819 (4)	0.142 (2)	
H18A	0.4882	0.9450	0.1511	0.213*	
H18B	0.4811	1.0694	0.1320	0.213*	
H18C	0.4156	1.0190	0.2237	0.213*	
C19	0.5982 (5)	1.1416 (4)	0.2928 (4)	0.1272 (19)	
H19A	0.5862	1.1959	0.2432	0.191*	
H19B	0.6737	1.1539	0.3257	0.191*	
H19C	0.5336	1.1447	0.3400	0.191*	
C20	0.6831 (6)	0.7415 (4)	0.5839 (4)	0.1226 (19)	
H20A	0.6209	0.7401	0.6332	0.184*	
H20B	0.7604	0.7479	0.6154	0.184*	
H20C	0.6805	0.6761	0.5461	0.184*	

### Atomic displacement parameters (Å^2^)

**Table:**

	*U*^11^	*U*^22^	*U*^33^	*U*^12^	*U*^13^	*U*^23^
O1	0.0560 (13)	0.0657 (14)	0.0828 (15)	−0.0095 (11)	0.0147 (13)	0.0056 (12)
O2	0.103 (2)	0.0614 (14)	0.0946 (18)	−0.0128 (13)	0.0082 (17)	−0.0075 (14)
O3	0.0623 (15)	0.122 (2)	0.0708 (15)	−0.0146 (15)	0.0000 (13)	−0.0310 (15)
O4	0.0420 (11)	0.0798 (14)	0.0556 (12)	−0.0051 (10)	0.0088 (10)	−0.0100 (11)
C1	0.0418 (16)	0.073 (2)	0.0635 (19)	0.0067 (15)	0.0045 (15)	0.0122 (17)
C2	0.060 (2)	0.097 (3)	0.087 (3)	0.012 (2)	0.026 (2)	0.012 (2)
C3	0.070 (2)	0.125 (3)	0.084 (3)	0.028 (3)	0.032 (2)	0.011 (3)
C4	0.095 (3)	0.103 (3)	0.081 (3)	0.029 (3)	0.011 (3)	−0.019 (2)
C5	0.083 (3)	0.070 (2)	0.075 (2)	0.008 (2)	0.010 (2)	−0.0050 (19)
C6	0.0466 (16)	0.0606 (17)	0.0564 (17)	0.0036 (14)	0.0032 (15)	0.0057 (15)
C7	0.0450 (16)	0.0618 (17)	0.0572 (16)	−0.0066 (14)	−0.0003 (15)	0.0057 (14)
C8	0.0407 (15)	0.0583 (17)	0.0533 (15)	−0.0003 (13)	−0.0028 (14)	0.0008 (14)
C9	0.0510 (18)	0.064 (2)	0.069 (2)	−0.0023 (15)	−0.0058 (16)	0.0037 (17)
C10	0.0445 (17)	0.0688 (19)	0.0579 (18)	0.0017 (15)	−0.0032 (15)	−0.0072 (16)
C11	0.0494 (17)	0.0660 (18)	0.0541 (17)	0.0028 (14)	0.0092 (15)	−0.0053 (15)
C12	0.0461 (17)	0.083 (2)	0.068 (2)	0.0034 (17)	0.0045 (16)	−0.0060 (18)
C13	0.0520 (19)	0.105 (3)	0.093 (3)	0.001 (2)	0.016 (2)	−0.008 (2)
C14	0.075 (3)	0.095 (3)	0.086 (3)	−0.019 (2)	0.036 (2)	−0.015 (2)
C15	0.082 (3)	0.079 (2)	0.066 (2)	−0.004 (2)	0.020 (2)	−0.0009 (19)
C16	0.063 (2)	0.074 (2)	0.0637 (19)	0.0068 (17)	0.0091 (17)	0.0003 (18)
C17	0.062 (2)	0.113 (3)	0.091 (3)	0.016 (2)	−0.002 (2)	0.019 (2)
C18	0.107 (4)	0.188 (6)	0.130 (4)	0.018 (4)	−0.044 (4)	0.027 (4)
C19	0.134 (4)	0.099 (3)	0.149 (4)	0.025 (3)	−0.001 (4)	0.023 (4)
C20	0.148 (5)	0.121 (4)	0.099 (3)	0.001 (4)	0.038 (4)	0.033 (3)

### Geometric parameters (Å, °)

**Table:**

O1—C1	1.370 (4)	C12—C13	1.540 (5)
O1—C9	1.374 (4)	C12—H12A	0.9800
O2—C9	1.199 (4)	C13—C14	1.512 (6)
O3—C10	1.201 (4)	C13—H13A	0.9700
O4—C10	1.328 (4)	C13—H13B	0.9700
O4—C11	1.472 (4)	C14—C15	1.504 (6)
C1—C2	1.374 (5)	C14—H14A	0.9700
C1—C6	1.392 (4)	C14—H14B	0.9700
C2—C3	1.363 (6)	C15—C20	1.525 (6)
C2—H2A	0.9300	C15—C16	1.535 (5)
C3—C4	1.389 (6)	C15—H15A	0.9800
C3—H3A	0.9300	C16—H16A	0.9700
C4—C5	1.382 (5)	C16—H16B	0.9700
C4—H4A	0.9300	C17—C19	1.517 (7)
C5—C6	1.396 (5)	C17—C18	1.537 (7)
C5—H5A	0.9300	C17—H17A	0.9800
C6—C7	1.434 (4)	C18—H18A	0.9600
C7—C8	1.338 (4)	C18—H18B	0.9600
C7—H7A	0.9300	C18—H18C	0.9600
C8—C9	1.463 (4)	C19—H19A	0.9600
C8—C10	1.485 (4)	C19—H19B	0.9600
C11—C16	1.508 (5)	C19—H19C	0.9600
C11—C12	1.522 (4)	C20—H20A	0.9600
C11—H11A	0.9800	C20—H20B	0.9600
C12—C17	1.539 (5)	C20—H20C	0.9600
			
C1—O1—C9	122.7 (2)	C12—C13—H13A	109.2
C10—O4—C11	117.9 (2)	C14—C13—H13B	109.2
O1—C1—C2	117.1 (3)	C12—C13—H13B	109.2
O1—C1—C6	120.9 (3)	H13A—C13—H13B	107.9
C2—C1—C6	121.9 (3)	C15—C14—C13	112.6 (3)
C3—C2—C1	119.2 (4)	C15—C14—H14A	109.1
C3—C2—H2A	120.4	C13—C14—H14A	109.1
C1—C2—H2A	120.4	C15—C14—H14B	109.1
C2—C3—C4	120.8 (4)	C13—C14—H14B	109.1
C2—C3—H3A	119.6	H14A—C14—H14B	107.8
C4—C3—H3A	119.6	C14—C15—C20	112.8 (4)
C5—C4—C3	119.9 (4)	C14—C15—C16	109.4 (3)
C5—C4—H4A	120.1	C20—C15—C16	110.9 (4)
C3—C4—H4A	120.1	C14—C15—H15A	107.9
C4—C5—C6	120.1 (4)	C20—C15—H15A	107.9
C4—C5—H5A	120.0	C16—C15—H15A	107.9
C6—C5—H5A	120.0	C11—C16—C15	111.7 (3)
C1—C6—C5	118.1 (3)	C11—C16—H16A	109.3
C1—C6—C7	117.6 (3)	C15—C16—H16A	109.3
C5—C6—C7	124.3 (3)	C11—C16—H16B	109.3
C8—C7—C6	121.5 (3)	C15—C16—H16B	109.3
C8—C7—H7A	119.2	H16A—C16—H16B	107.9
C6—C7—H7A	119.2	C19—C17—C18	110.4 (4)
C7—C8—C9	120.4 (3)	C19—C17—C12	114.0 (4)
C7—C8—C10	122.4 (3)	C18—C17—C12	111.3 (4)
C9—C8—C10	117.2 (3)	C19—C17—H17A	106.9
O2—C9—O1	116.6 (3)	C18—C17—H17A	106.9
O2—C9—C8	126.4 (3)	C12—C17—H17A	106.9
O1—C9—C8	116.9 (3)	C17—C18—H18A	109.5
O3—C10—O4	124.8 (3)	C17—C18—H18B	109.5
O3—C10—C8	124.3 (3)	H18A—C18—H18B	109.5
O4—C10—C8	110.9 (3)	C17—C18—H18C	109.5
O4—C11—C16	108.8 (2)	H18A—C18—H18C	109.5
O4—C11—C12	106.2 (2)	H18B—C18—H18C	109.5
C16—C11—C12	114.1 (3)	C17—C19—H19A	109.5
O4—C11—H11A	109.2	C17—C19—H19B	109.5
C16—C11—H11A	109.2	H19A—C19—H19B	109.5
C12—C11—H11A	109.2	C17—C19—H19C	109.5
C11—C12—C17	114.3 (3)	H19A—C19—H19C	109.5
C11—C12—C13	108.3 (3)	H19B—C19—H19C	109.5
C17—C12—C13	113.6 (3)	C15—C20—H20A	109.5
C11—C12—H12A	106.7	C15—C20—H20B	109.5
C17—C12—H12A	106.7	H20A—C20—H20B	109.5
C13—C12—H12A	106.7	C15—C20—H20C	109.5
C14—C13—C12	112.1 (3)	H20A—C20—H20C	109.5
C14—C13—H13A	109.2	H20B—C20—H20C	109.5
			
C9—O1—C1—C2	176.7 (3)	C11—O4—C10—C8	177.2 (2)
C9—O1—C1—C6	−1.0 (4)	C7—C8—C10—O3	133.2 (4)
O1—C1—C2—C3	−177.6 (3)	C9—C8—C10—O3	−44.8 (5)
C6—C1—C2—C3	0.1 (5)	C7—C8—C10—O4	−44.5 (4)
C1—C2—C3—C4	−0.8 (6)	C9—C8—C10—O4	137.5 (3)
C2—C3—C4—C5	0.7 (7)	C10—O4—C11—C16	−83.0 (3)
C3—C4—C5—C6	0.1 (6)	C10—O4—C11—C12	153.7 (3)
O1—C1—C6—C5	178.3 (3)	O4—C11—C12—C17	−58.8 (4)
C2—C1—C6—C5	0.7 (5)	C16—C11—C12—C17	−178.7 (3)
O1—C1—C6—C7	−1.3 (4)	O4—C11—C12—C13	173.5 (3)
C2—C1—C6—C7	−178.9 (3)	C16—C11—C12—C13	53.6 (4)
C4—C5—C6—C1	−0.8 (5)	C11—C12—C13—C14	−53.9 (4)
C4—C5—C6—C7	178.8 (3)	C17—C12—C13—C14	178.0 (3)
C1—C6—C7—C8	1.6 (4)	C12—C13—C14—C15	57.7 (4)
C5—C6—C7—C8	−178.0 (3)	C13—C14—C15—C20	−179.8 (3)
C6—C7—C8—C9	0.3 (4)	C13—C14—C15—C16	−55.8 (4)
C6—C7—C8—C10	−177.6 (3)	O4—C11—C16—C15	−173.5 (3)
C1—O1—C9—O2	−175.3 (3)	C12—C11—C16—C15	−55.1 (4)
C1—O1—C9—C8	2.9 (4)	C14—C15—C16—C11	53.8 (4)
C7—C8—C9—O2	175.4 (3)	C20—C15—C16—C11	178.8 (4)
C10—C8—C9—O2	−6.6 (5)	C11—C12—C17—C19	−66.3 (5)
C7—C8—C9—O1	−2.5 (4)	C13—C12—C17—C19	58.5 (5)
C10—C8—C9—O1	175.5 (2)	C11—C12—C17—C18	167.9 (4)
C11—O4—C10—O3	−0.5 (5)	C13—C12—C17—C18	−67.2 (5)

### Hydrogen-bond geometry (Å, °)

**Table:**

*D*—H···*A*	*D*—H	H···*A*	*D*···*A*	*D*—H···*A*
C3—H3A···O2^i^	0.93	2.43	3.288 (5)	154
C5—H5A···O3^ii^	0.93	2.42	3.276 (5)	152

Symmetry codes: (i) −*x*+5/2, −*y*+2, *z*−1/2; (ii) −*x*+2, *y*−1/2, −*z*+1/2.
